# Microparticle manipulation using laser-induced thermophoresis and thermal convection flow

**DOI:** 10.1038/s41598-020-76209-9

**Published:** 2020-11-05

**Authors:** Yang Qian, Steven L. Neale, John H. Marsh

**Affiliations:** James Watt School of Engineering, University of Glasgow, Glasgow, G12 8QQ UK

**Keywords:** Lab-on-a-chip, Optical manipulation and tweezers

## Abstract

We demonstrate manipulation of microbeads with diameters from 1.5 to 10 µm and Jurkat cells within a thin fluidic device using the combined effect of thermophoresis and thermal convection. The heat flow is induced by localized absorption of laser light by a cluster of single walled carbon nanotubes, with no requirement for a treated substrate. Characterization of the system shows the speed of particle motion increases with optical power absorption and is also affected by particle size and corresponding particle suspension height within the fluid. Further analysis shows that the thermophoretic mobility (*D*_*T*_) is thermophobic in sign and increases linearly with particle diameter, reaching a value of 8 µm^2^ s^−1^ K^−1^ for a 10 µm polystyrene bead.

## Introduction

Over recent decades micromanipulation has become a useful tool for studies in the life science and nanophysics, including cell sorting^1,2^, cell motility^3^, protein immunoassays^4,5^, molecular chemistry^6,7^, intracellular interaction^8,9^ and compositions of particles^10^. Several platforms for microparticle manipulation have been developed, each having their own advantages and limitations and suitable for different experimental conditions and applications. In optical approaches, optical tweezers use the optical gradient force induced by a tightly focused laser beam to trap and move particles^11,12^, however, a complex optical system aligned with high accuracy is required. Optoelectronic tweezers use lower light intensities than optical tweezers but require specific electrical properties of the samples and devices^13–15^. In electrical approaches, electrokinetics^16^ and dielectrophoresis (DEP)^17^ require an electric field to be established in the fluid to move particles, however both techniques place restrictions on the electrical conductivity of the buffer ^16–18^. Other approaches, such as hydrodynamic flow^18,19^, magnetic tweezers^20,21^ and acoustic tweezers^22–24^, are not capable of producing arbitrary patterns of microparticles.

Thermophoresis, or the Soret effect, is a phenomenon in which a mixture of suspended particles migrates along a thermal gradient (∇*T*) established in the system. In a liquid experiencing no overall net flow, thermophoresis can be considered as a particle transport mechanism arising from a temperature gradient superimposed on Brownian diffusion. For a colloidal sample with a low concentration (*c*) of particles in the presence of a thermal gradient (∇*T*), the total mass flux (*J*) can be written as^25,26^

1 $$ J = J_{D} + J_{TD} = ( - D\nabla c) + ( - cD_{T} \nabla T) $$

where *D* is the Brownian diffusion coefficient, ∇*c* is the mass concentration gradient of particles, and *D*_*T*_ is the thermal diffusion coefficient (also termed the thermophoretic mobility). The first term, *J*_*D*_, arises from Brownian diffusion based on Fick’s first law, and the second term, *J*_*TD*_, arises from thermal diffusion^27,28^. Since mass flux can also be expressed as the product of the velocity field of flowing mass elements and the mass concentration (*c*), the thermophoretic velocity (*v*_*th*_) can be written as^26,29,30^

2 $$ v_{th} = - D_{T} \nabla T. $$

In the steady state, thermophoretic transport and Brownian diffusion are balanced (*J* = 0), so for small temperature differences (*dT*), we obtain

3 $$ \frac{dc}{c} = - S_{T} dT $$

where *S*_*T*_ is Soret coefficient and *S*_*T*_ = *D*_*T*_/*D*. The particle transport direction is determined by the sign of *D*_*T*_. When *D*_*T*_ > 0, particles move towards a colder region with ‘thermophobic’ behaviour, whereas particles move towards a warmer region with ‘thermophilic’ behaviour when *D*_*T*_ is negative^26,29,31^. Unlike in gases where there are well-developed models of thermophoresis, there is no general physical model of particle thermophoresis in liquids, nor fundamental understanding of the characteristics of *D*_*T*_. As a result, the applications of thermophoresis in colloidal science have been limited.

Several researchers have studied the dependence of *D*_*T*_ on the ionic strength^32,33^, pH^32,34^ and temperature^34–37^ of the sample solution. However, there is no consensus about the effect of particle size, and so the conclusions are still controversial^30,32,36,38^. Regarding ‘thermophilic’ and ‘thermophobic’ behaviour, particles have been trapped within a localized hot region^39–41^, confined within dynamic inhomogeneous temperature fields^42–44^ and simply diverted^45^. However, the temperature gradient is generated in most cases by pre-treating the surface of the substrate.

**Figure 1:**
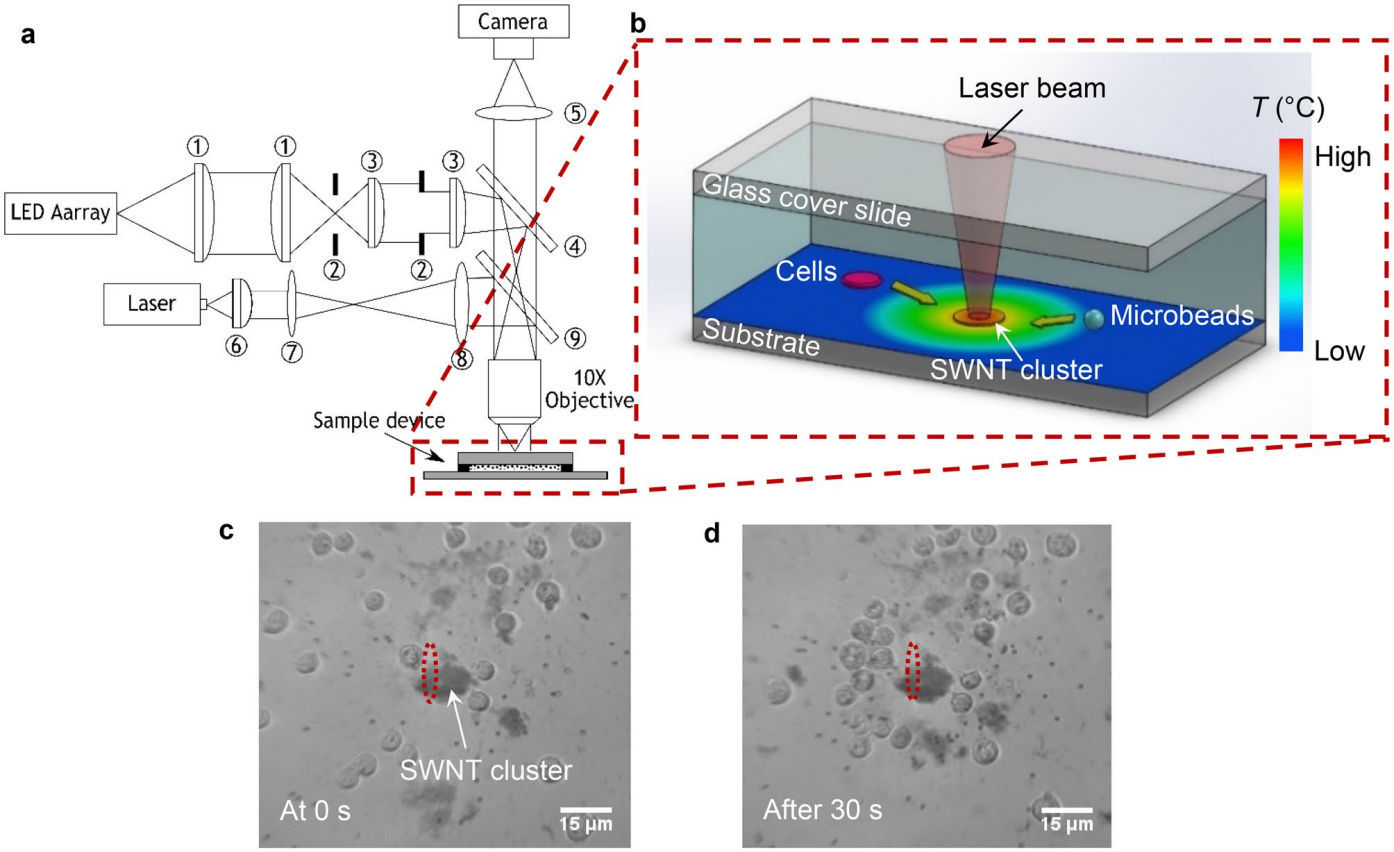
Experimental setup and demonstration of manipulation of cells and microbeads using optical-thermophoretic tweezing. (**a**) Schematic of the experimental setup; ① and ③: aspheric condenser lenses with different focal lengths, ②: condenser/field diaphragm, ④: 50:50 beamsplitter, ⑤: tube lens, ⑥: aspheric collimation lens, ⑦ and ⑧: bi-convex lenses with different focal lengths, ⑨: short pass dichroic filter. (This schematic was created by Inkscape v0.91, URL: https://inkscape.org/). (**b**) 3D schematics of the sample device and the microparticle motion in a localized temperature field established by laser heating on a SWNT cluster. The actual dimensions of the fluid volume inside the chamber were 20 mm (*L*) × 13 mm (*W*) × 100 µm (*H*) (this schematic was created by SolidWorks v2019, URL: https://www.solidworks.com/). (**c**,**d**) Image stack at 0 s and after 30 s, showing microparticle manipulation by a laser illuminated SWNT cluster (for full record, see Supplementary Video S3). Cells and microbeads were trapped and confined in a ring-shaped region around the cluster. Laser spot size (highlighted by the red dashed ovals): *Φ*2.5 µm in the *x*-direction, *Φ*52.9 µm in the *y*-direction.

In this study, we successfully exploit laser-induced thermophoresis and thermal convection flow to achieve particle manipulation in a disposable and biocompatible fluidic device with a non-treated substrate. A temperature distribution is established within the device by absorbing laser light in a cluster of single walled carbon nanotubes (SWNTs) suspended in the sample solution. The temperature gradient establishes a convection flow in the fluid and results in thermophoretic forces acting on the particles. The combination of the convection flow and thermophoresis results in microparticles (i.e. both microbeads and cells) becoming trapped and confined within a ring-shaped region around the localized hot region (see Fig. 1c,d), achieving ‘optical-thermophoretic tweezing’. Although similar phenomena have been reported by other research groups^46–52^, in many cases the substrates of their devices needed to be pre-treated by a thin layer of high optical absorber, and in no cases have biological cells been used. Our method of using SWNTs clusters instead of an absorbing substrate is important as it will allow cells to be concentrated within standard glass/plastic-ware such as slides, well plates and Petri dishes without modification, which significantly reduces the device fabrication time and cost and so is truly suitable for single-use applications. As well as demonstrating manipulation of microbeads and cells, we have characterized the physics by investigating the particle speeds and trajectories under different incident optical powers and for a range of particle sizes. This paper reports both experimental studies and numerical modelling.

In contrast to tweezing techniques based on direct optical manipulation, our system uses only a CW diode laser and lenses with low numerical aperture (NA), providing a compact, simple and low-cost optical platform with a low optical intensity. The sample device is cheap, easy to assemble and use. Our sample solutions are straightforward to prepare and do not require tailored physical properties, for example the restrictions placed on electrical conductivity by electrokinetics and DEP identified above. Our platform allows microbeads and cells to be concentrated with very little modification of standard protocols; it is therefore a flexible platform for micromanipulation, catering for applications such as microparticle sorting. It therefore has the potential to become an important and versatile tool in the life science field.

## Methods

### Optical system setup

A schematic of the optical system setup used in the experiments is shown in Fig. 1a. The whole setup comprised two systems: a reflected light microscope system and a semiconductor laser projection system. A single 10× objective (NA = 0.25) was shared by the two systems to project both the illumination beam and the laser beam onto samples. The microscope system was designed based on the Köhler illumination technique, aiming to create a smooth and uniform illumination background. In order to achieve this, the image of the light source needs to be defocused after the objective and projected onto the sample focal plane. Therefore, for the illumination path, a cold white LED array was used as the illumination source, followed by two groups of aspheric condenser lenses, labelled ① and ③ with different focal lengths. The purpose of the first lens group was to create a second light source to enhance the overall illumination. The second lens group was to create an image of the second light source at the rear focal plane of the objective, meanwhile adjusting the size of the image to ensure the rear aperture of the objective was fully illuminated. The two irises ② were used as the condenser diaphragm and the field diaphragm, respectively. The condenser diaphragm was used to control the brightness and contrast of the illumination background. The field diaphragm was to adjust the size of the illumination background. The beam after the condenser lens was reflected by a 50:50 beamsplitter ④ and projected into the objective. For the imaging path, a tube lens ⑤ and a CMOS camera with resolution of 1280 × 1024 pixels (DCC1645C, Thorlabs, Inc) were used.

In terms of the laser system, a multimode CW laser diode with wavelength of 808 nm and maximum optical power of 1 W was chosen, followed by an aspheric collimation lens ⑥ with short focal length used to collect and collimate the laser beam. A beam expander constructed from two bi-convex lenses (⑦ and ⑧) with different focal lengths and a short pass dichroic filter ⑨ were then added into the optical path to ensure the laser beam was fully reflected into the objective and filled its entire rear aperture, while being transparent to visible light.

In the experiments, the incident optical power was kept below 180 mW to avoid boiling the water, due to the high optical absorption of the SWNT cluster. The size of the spot focused on the cluster was *Φ*2.5 µm in the *x*- and *Φ*52.9 µm in the *y*-direction. The experiments were recorded at a video frame rate of 20 fps for 30 s. The responses of beads of interest were analysed by particle tracking software (Tracker v4.11.0, Douglas Brown).

### Device fabrication

The sample device was a thin fluidic chamber constructed by attaching a glass cover slide, cut to appropriate dimensions, onto a normal microscope slide separated by a thin spacer frame (see Fig. 1b). Here, the dimensions of the fluid volume inside the chamber were 20 mm (*L*) × 13 mm (*W*) × 100 µm (*H*). Two holes, with diameter of 2 mm drilled diagonally and close to diagonally opposite corners of the cover slide, were used as the inlet and outlet. After injecting the sample solution into the chamber, the inlet and the outlet were sealed by tape. The exterior edges of the chamber were sealed by a small amount of petroleum jelly to prevent perturbations from evaporation. The thickness of the chamber was around 100 µm. The dimensions (*L* × *W* × *H*) of the glass cover slide and the microscope slide were 26 mm × 19 mm × 1 mm and 76 mm × 26 mm × 1 mm respectively. For the experiment on biological cell manipulation, the device was sterilized under UV light exposure for 2 h before implementing the experiment^53^.

### Sample solution preparation

The colloidal mixture of microbeads and SWNT clusters was prepared by dispersing a pre-washed SWNT conductive ink into fresh cell culture medium RPMI1640 (Gibco, Thermo Fisher Scientific Inc.) at ratio of 140 mg in 1 mL followed by vortex mixing. To allow appropriate clusters to form, the suspension was then sonicated for 2 h in a beaker cooled with ice cubes. The as-supplied polystyrene microbead stock solutions (10% w/v for each, Bangs Laboratories Inc.) with different bead sizes (1.54 µm, 2.88 µm, 4.95 µm, 7.79 µm and 9.97 µm in diameter) were suspended in the SWNT cluster suspension with dilution ratios of 0.5 µL:1 mL, 3 µL:1 mL, 0.5 µL:1 mL, 20 µL:1 mL and 60 µL:1 mL, followed by vortex mixing. Here, the as-supplied SWNT conductive ink (1 mg/mL, Sigma-Aldrich Co. LLC) was washed with ultra-pure deionized water by centrifugation for three times to remove any solvent residues. The RPMI1640 culture medium was chosen, as the final purpose of the experiment was to manipulate biological cells.

For the biological mixture, a colloidal mixture of microbeads and SWNT clusters was sterilized under UV light for 1 h, followed by adding a Jurkat cell solution with pre-adjusted concentration (Jurkat, Clone E6­1 (ATCC TIB-152), 9.15 × 10^7^ cells/mL) with a dilution ratio of 20 µL:1 mL. Note that once the sample solution was sterilized, the rest of the preparation was performed under a cell culture hood.

## Results and discussion

As shown in Fig. 1b, the localized thermal gradient was established by focusing a CW diode laser beam (808 nm) onto a cluster of SWNTs suspended in a sample solution that also contained either polystyrene microbeads (diameter ranging from 1.5 to 10 µm) or a colloidal mixture of such microbeads and Jurkat cells.

**Figure 2:**
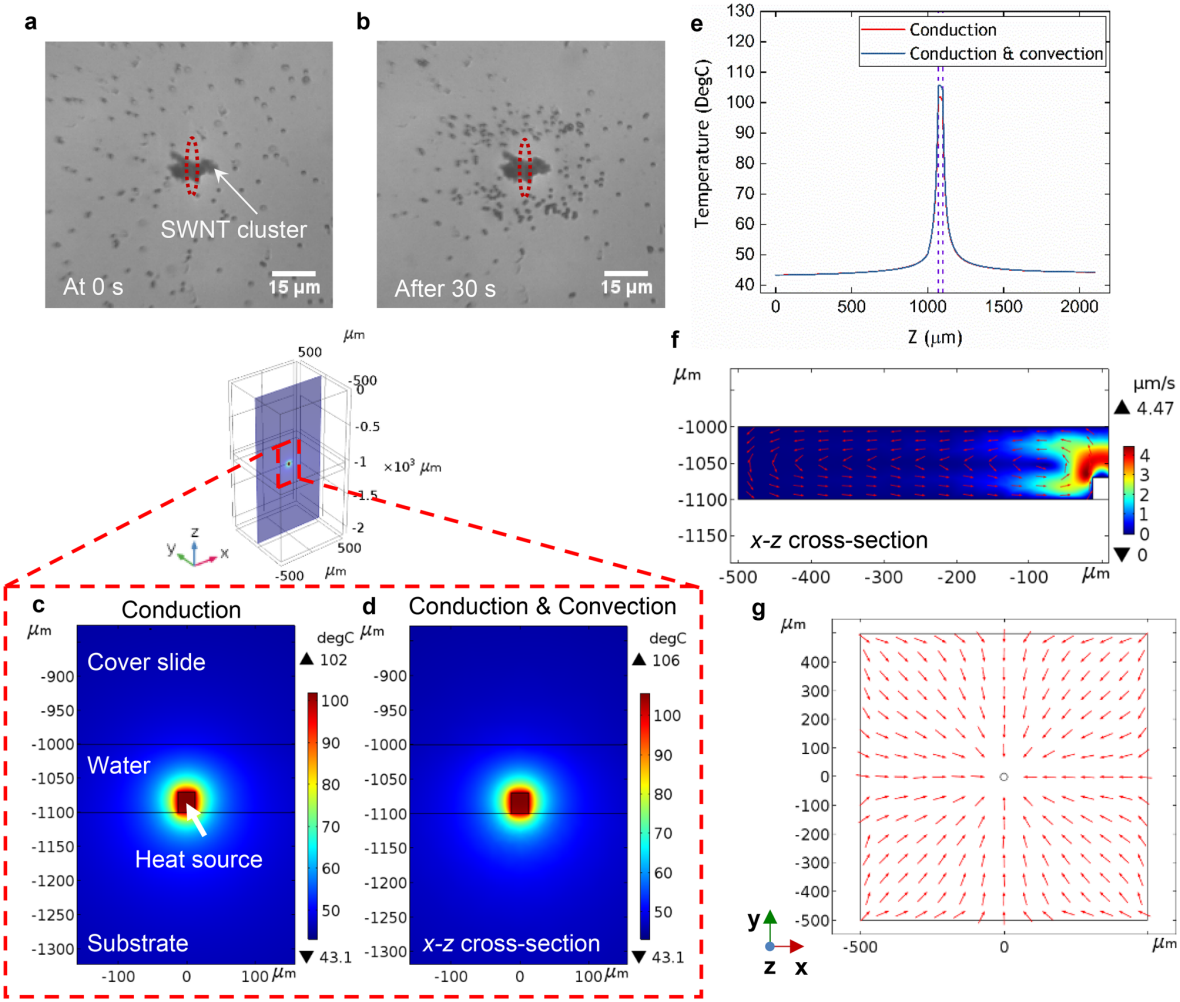
Demonstration of manipulation of microbeads and related numerical modelling. (**a**,**b**) Positions of 1.54 µm polystyrene beads using SWNT cluster on glass substrate system at 0 s and after 30 s using incident laser power of 180 mW (for full record, see Supplementary Video S1). Laser spot size (highlighted by the red dashed ovals): *Φ*2.5 µm in the *x*-direction, *Φ*52.9 µm in the *y*-direction. (**c**,**d**) Numerical modelling of temperature distributions within the 3D structure in *x–z* cross-section for different types of heat transfer: conduction only and combination of conduction and convection. (**e**) Comparison of modelled temperature distributions along the geometrical centre of the structure in *z*-axis under the same types of heat transfer as (**c**,**d**). The region highlighted by the dashed lines represents the heat source. (**f**) Modelled fluid flow velocity magnitude distributions (shown as colour background) and directions (red arrows) in *x–z* cross-section of the water domain. (**g**) Plan view of modelled directions of fluid flow in an *x–y* plane just above the substrate.

In the first experiment, a colloidal solution of SWNT clusters and polystyrene microbeads with diameter of 1.54 µm was used as the sample solution. We found that most particles moved towards the beam, stopping at a certain distance from the illuminated cluster and leaving a clear region around it. The obvious ring shape formed by the particles is shown in Fig. 2a,b (also see Supplementary Video S1 for a real-time video). However, by varying the focal plane of the microscope it was observed that a small number of particles at different heights in the fluid exhibited different behaviour: some particles close to the substrate moved towards the SWNT cluster, were redirected upwards as they approached the cluster, and then moved away from the cluster. After travelling a certain distance, the microbeads moved down towards the substrate and repeated this pattern continuously (see Supplementary Video S2 for a real-time video). This phenomenon confirms the presence of a laser-induced thermal convection flow (see Fig. 2f). The different movement of this small number of particles was ascribed to them being at different suspension heights to the majority of particles.

The ring-shaped region was formed by particles experiencing two balanced forces: thermophoresis, pushing particles away from the hot region, and thermal convection flow, dragging particles towards the SWNT cluster. In almost all cases, once the beads reached an equilibrium position they remained in focus, which demonstrated any vertical component of the Stokes drag force was weaker than the gravity force. Some of the stopped particles exhibited random movements around their equilibrium positions, others appeared to be more completely immobilised and some formed loose clusters. It is possible electrostatic forces may be modified by the increased temperature close to the illuminated cluster, causing some particles to be attracted to each other and to the substrate surface—this needs to be verified by further investigation. Photophoresis could be eliminated as a significant force in this experiment because almost no beads were illuminated by the laser beam, as seen in Fig. 2a.

The laser-induced thermal convection flow was studied by numerical modelling using COMSOL. The modelling was implemented by coupling two physical models: the heat transfer model and the incompressible laminar flow model. The temperature distribution within the sample device under different types of heat transfer was studied first. As shown in Fig. 2c–e, there are no obvious differences in temperature distribution for thermal conduction only and a combination of thermal conduction and convection, which is consistent with the low value (1.14) of the theoretical calculation of the Rayleigh number^54^. The region between the two dashed lines in Fig. 2e represents the temperature distribution within the heat source (SWNT cluster). The clear conclusion is heat flow due to conduction dominates that due to convection. The temperature decreases with distance from the heat source (SWNT cluster) COMSOL domain in all three dimensions. The temperatures inside the liquid around the cluster are close to 100 °C, which agrees with the real experiments—in a few cases bubbles were seen, suggesting the temperatures around illuminated clusters could be approaching the boiling point of water.

Second, the fluid flow that arose from the localized temperature gradient under the steady-state condition was modelled. As shown in Fig. 2f, a loop of convection fluid flow is created, as illustrated by the red arrows. Figure 2g shows a plan view of the flow directions in an *x–y* plane slightly above the substrate surface, i.e. just above the boundary layer. Obviously, symmetrical convection flows are formed in all three dimensions.

A control experiment without any SWNT clusters was also implemented. Unexpectedly, we found that particles still moved towards the light spot to form a ring-shaped stopping area, although at a considerably lower velocity. To investigate this phenomenon, we compared experimental and modelled particle velocities with and without SWNT clusters. The COMSOL model took account of heat generation from absorption of the laser beam within the glass cover slide, the fluid chamber and the glass substrate. At a wavelength of 808 nm, the absorption coefficient of water is 0.0223 cm^−1^ and of soda-lime glass is 0.403 cm^−1^^55^, indicating optical absorption within the soda-lime glass is the dominant source of heat. The Fresnel reflectance at normal incidence at the water–glass interface was small (4.36 × 10^−3^) and could be neglected.

The model for the control experiment shows that a symmetrical convection flow is created inside the fluid chamber (see Supplementary Fig. S1 and Fig. S2), similar to when an SWNT is present (Fig. 2f). The values of the experimental and modelled velocities 75 µm away from the laser beam are compared in Table 1. The first point to note is the agreement between the experiment and modelled data is surprisingly close, given the COMSOL model uses physical parameters taken only from the literature, including, for example, the absorption coefficients of water and glass which have some uncertainty. The velocity predicted by the numerical model is only a factor of 1.5 lower than experiment for the situation with SWNT clusters and 1.7 for the control experiment. The second point is that the ratios of the velocities with and without SWNT clusters are also close for experiment (35) and the model (40).

**Table 1:** The average particle velocity 75 µm from the beam centre for the experiment with SWNT clusters and the control experiment without SWNT clusters for both experimental and modelled data.

	Average particle speed with SWNT clusters (µm s^−1^)	Average particle speed without SWNT clusters (µm s^−1^)
Experiment	1.4	0.04
Modelled	0.92	0.023
Ratio	1.5	1.7

### Theoretical model

An analytic model of the system was developed to identify the underlying physics and was then used as the basis for further data analysis. When an SWNT cluster is illuminated, particles initially move towards the cluster, then slow down and stop (Fig. 2a,b). This behaviour suggests the particles are responding to forces arising from (at least) two distinct physical phenomena with different dependencies on distance. We identify these phenomena as the Stokes drag force arising from convection flow and thermophoresis arising from the conduction of heat.

**Figure 3:**
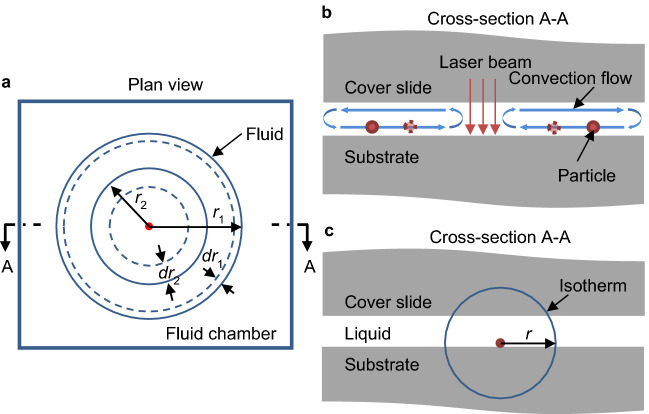
Theoretical model of the underlying physics. (**a**,**b**) Simplified physical model of 2D fluid convection flow in the fluid chamber in plan and cross-section (A-A) views. (**c**) Theoretical model of heat transfer within the sample device viewed in cross-section A-A.

The dimensions of the chamber shown in Fig. 1b are ~ 100 µm high and around 20 mm (*L*) × 13 mm (*W*) in area. Apart from in the region close to the heated cluster and at the edges of the chamber, the convection flow can be viewed as laminar fluid flow in 2D, in towards the heated region in the lower half of the chamber (i.e. for heights < 50 µm) and outwards in the upper half of the chamber. To analyse the flow we consider a thin layer of fluid of vertical thickness *δd* in the lower half of the chamber at a radius *r*_1_ from the centre of the cluster, as shown in Fig. 3a,b. In a time *dt*, the fluid moves a distance *dr*_1_, and so the volume of fluid crossing the circle described by *r*_1_ is 2*πr*_1_*dr*_1_·*δd*. At a radius *r*_2_ closer to the heated region, the fluid moves a distance *dr*_2_ in the same *dt* with an associated volume 2*πr*_2_*dr*_2_·*δd*. Equating these two volumes and dividing by *dt*, we obtain:

4 $$ 2\pi r_{1} \cdot \frac{{dr_{1} }}{dt} = 2\pi r_{2} \cdot \frac{{dr_{2} }}{dt} \Rightarrow r_{1} \cdot v_{1} \left( r \right) = r_{2} \cdot v_{2} \left( r \right) = - a \Rightarrow v_{FLOW} \left( r \right) = - \frac{a}{r} $$

where, *a* is a constant and *v*(*r*) represents the flow velocity. *v*(*r*) is therefore proportional to 1/*r*, with the negative sign indicating the flow is towards the heat source.

The thermophoretic velocity is given by Eq. (2). The thermal gradient can be found by considering the cluster as a point heat source. Heat transfer is predominantly by conduction even in the water domain because of the low Rayleigh number. The glass substrate and cover are relatively thick and the thermal conductivities of glass (0.8 W m^−1^ K^−1^) and water (0.6 W m^−1^ K^−1^) are similar. The isotherms are therefore to first order spherical in shape and centred on the heat source, as shown in Fig. 3c.

Fourier’s law^56^ is

5 $$ q = - k\nabla T $$

where *q* is the heat flux, *k* is the thermal conductivity and ∇*T* is the temperature gradient. Because the isotherms are approximately spherical, the gradient of the temperature $\nabla T=dT/dr$ and the total heat flow across an isotherm is given by *Q* = 4*πr*^2^*q*. We can therefore write:

6 $$ \nabla T = - \frac{Q}{4\pi k} \cdot \frac{1}{{r^{2} }} = - \frac{{b^{\prime}}}{{r^{2} }} $$

where *b*′ is a parameter proportional to *Q*, and *r* is the radius of a spherical isotherm. Substituting into Eq. (2), the velocity component arising from thermophoresis is:

7 $$ v_{TH} \left( r \right) = \frac{b}{{r^{2} }} $$

where $b = b^{\prime} \cdot D_{T}$. The total velocity, *v*_*p*_, is the sum of ${v}_{FLOW}\propto 1/r$ and ${v}_{TH}\propto 1/{r}^{2}$ and can be expressed as:

8 $$ \frac{dr}{{dt}} = v_{p} = v_{FLOW} \left( r \right) + v_{TH} \left( r \right) = - \frac{a}{r} + \frac{b}{{r^{2} }} $$

where *a* and *b* are both positive. Multiplying by *dt*, we obtain:

9 $$ dt = \frac{{r^{2} }}{b - ar} \cdot dr $$

and integrating gives:

10 $$ \int {dt} = \int {\frac{{r^{2} }}{b - ar} \cdot dr} \Rightarrow t = - \frac{{2b^{2} \ln \left( {b - ar} \right) + ar\left( {ar + 2b} \right)}}{{2a^{3} }} + C $$

where *C* is a constant of integration.

The working mechanisms can be summarized as follows: when the beam illuminates the SWNT cluster (or indeed is absorbed in the glass/water in the control experiment), light energy is absorbed and converted into heat establishing a temperature gradient within the device. Even though there is convection within the fluid, at the low Rayleigh number operating here heat transfer is dominated by thermal conduction through the entire device. The temperature distribution causes the fluid density to vary within the chamber; fluid with low density rises and is then pushed away from the hot region by further rising fluid. It then cools and falls under gravity at the edges of the chamber establishing the convection flow. The temperature gradient also causes a thermophoretic force to act on particles. Particles in the lower half of the fluid chamber therefore experience a Stokes force dragging them towards the SWNT cluster and a thermophoretic force pushing them away, their individual trajectories being the result from the combined action of these forces.

### Further investigation of particle-stopping position

**Figure 4:**
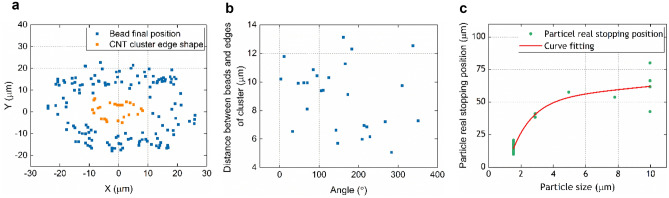
Investigation of the particle-stopping region. (**a**) Plots of the bead final positions and the SWNT cluster edge in the case of 1.54 µm beads. (**b**) The related distance plot between final bead positions and the cluster edge as a function of angle. (**c**) Experimental microbead-stopping position as a function of bead size.

To elucidate the characteristics of the convection flow and thermophoresis, the particle-stopping position (ring-shaped region) as a function of particle size was studied first. Figure 4a shows the microbead final positions and the edge of the SWNT cluster in the case of 1.54 µm beads, and Fig. 4b the related distance between the particles and the cluster edge as a function of the directional angle. Here, each position point on the edge of the cluster was the crossing point between a line drawn from the beam centre to the bead and the cluster edge. The beam centre is represented as (0, 0) in Fig. 4a, and the related directional angle was based on the position of the bead of interest and the positive *x*-axis. Figure 4b shows the data points are scattered over a range of 5 µm to 13 µm regardless of direction, which indicates that the bead-stopping positions are determined by the shape of the cluster rather than that of the (elliptically shaped) optical beam. In the model we therefore assume optical absorption results in a uniform heat distribution across the cluster. By analysing the particle-stopping position with different particle sizes, it was found that the distance at which beads stop from the cluster centre increased with bead size, as shown in Fig. 4c.

### Further investigation of particle speed

**Figure 5:**
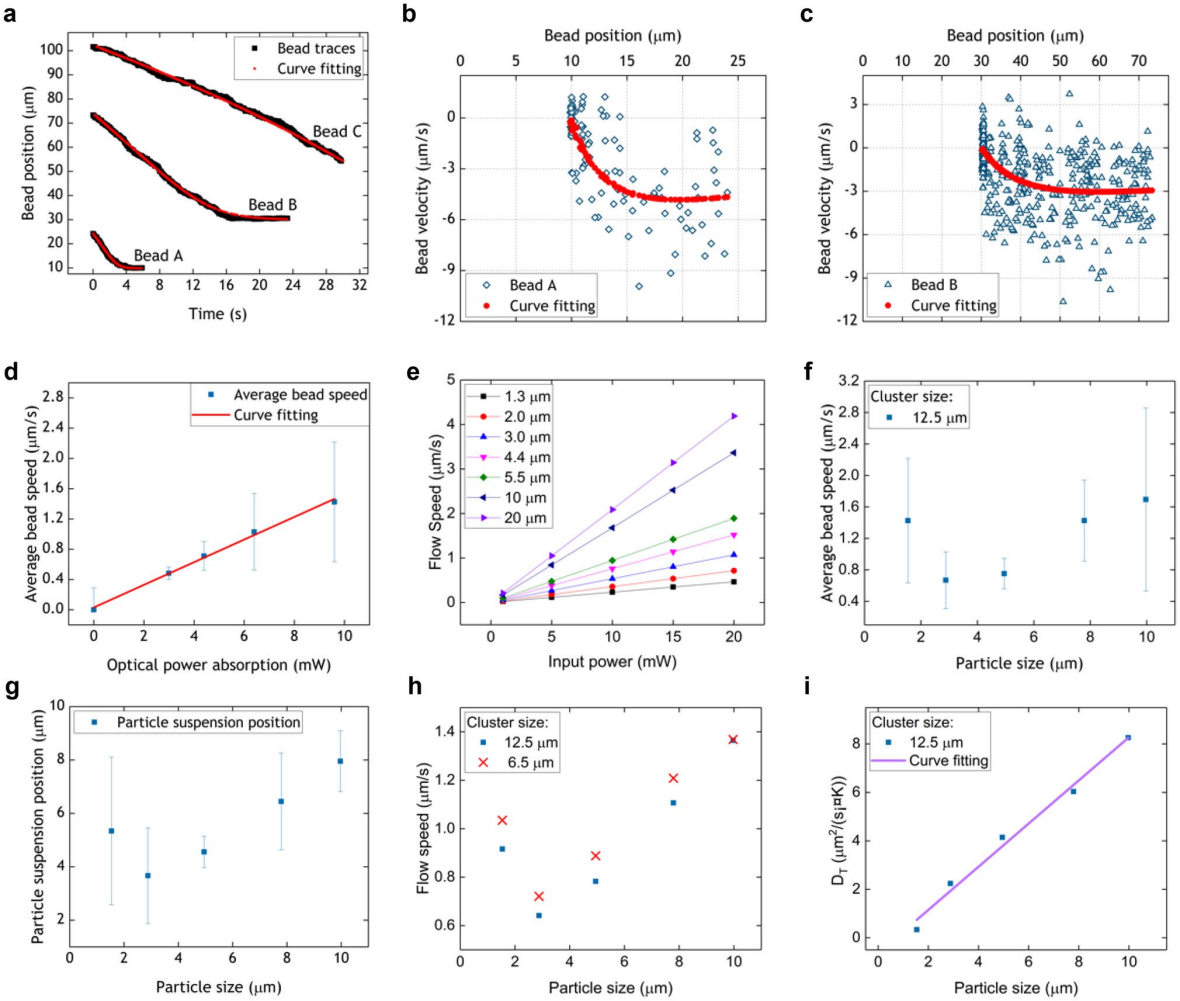
Studies of particle trajectory, speed, suspension height, and the thermophoretic mobility. (**a**) Plots of the position of three individual polystyrene microbeads (*Φ*1.54 µm) as a function of time, and related curves fitted based on Eq. (10). (**b**,**c**) Related bead velocity plots as a function of bead position along with curve fitting based on Eq. (8). (**d**) Average bead speed for different absorbed optical powers at a distance from the beam centre of 75 µm. (**e**) Modelled convection flow speed as a function of absorbed power 75 µm from the beam centre and at different heights above the substrate domain. (**f**) Plot of average bead speed as a function of bead size 75 µm from the beam centre at an optical power absorption of around 10 mW. (**g**) Plot of average particle suspension height as a function of particle size. (**h**) Modelled flow speed as a function of particle size based on the real particle suspension heights with different cluster sizes under the same optical power absorption (10 mW). (**i**) Plot of the thermophoretic mobility (*D*_*T*_) as a function of particle size for the same optical power absorption (10 mW) based on the average particle-stopping positions measured experimentally. The error bars shown in (**d**), (**f**) and (**g**) represent the standard deviations of the measurements.

Second, the particle speed was investigated. A relation between particle position and time, Eq. (10), has been derived based on our theoretical model. Figure 5a plots the trajectories of three typical beads with different starting positions, together with curves fitted using Eq. (10). Initially, the Stokes force from the convection flow is dominant which causes beads move towards the cluster. As they move in towards the heated cluster the thermophoretic force increases more rapidly and, when the forces balance, the particles stop moving. Although beads A and B in Fig. 5 stopped at different distances, this was due to particle accumulation stopping bead B at the exterior edge of the ring region. During the limited period of recording, bead C did not have time to reach the ring. Figure 5b,c show the velocity as a function of radial position for beads A and B, along with curves fitted using Eq. (8). Here, the values of *a* and *b* used in Eq. (8) were derived from the fitted curves in Fig. 5a. The good curve fittings shown in these three figures suggests that our theoretical model represents the real particle motion.

The dependence of the particle speed on absorbed optical power was also studied. The power absorbed by individual SWNT clusters was measured as follows. The device was placed on top of an optical power sensor (S130C, Thorlabs, Inc.), and the incident beam was set at five different power levels. For each power, measurements were taken in two regions of the sample: one region where the laser beam illuminated an SWNT cluster and one region without any clusters; the fraction of power absorbed by an individual cluster was then calculated. To ensure consistency, clusters were selected to be of similar shape and thickness as far as possible. This was done by (a) imaging their cross-sections in the *x–y* plane and (b) measuring their optical absorption to ensure they had similar thicknesses in the *z*-plane. Other parameters were as for the previous experiment.

To determine the parameters *a* and *b*, the trajectories of individual particles were curve fitted. Figure 5d shows a plot of the average bead speed 75 µm away from the beam centre as a function of absorbed optical power. The bead speed is proportional to the absorbed power, indicating the dominant Stokes drag force increases linearly with the generation of heat. It should be noted that when no power is absorbed by the SWNT cluster particles might be expected to be static, but random particle movements are always present because of Brownian motion, with the error bar at zero power reflecting this motion. Figure 5e displays the modelled convection flow speed as a function of input power at (*x, y*) = (75 µm, 0 µm) at different heights along the *z*-axis, i.e. heights above the substrate. For a constant height, the trend in flow speed agrees with the experimental results. Similarly, at a constant input power, the flow speed increases with height, indicating the importance of the particle suspension height.

Experiments were performed with five different sizes of bead (*Φ*1.54 µm, *Φ*2.88 µm, *Φ*4.95 µm, *Φ*7.79 µm and *Φ*9.97 µm). Again, the power absorption was monitored and SWNT clusters of similar shapes and thicknesses were chosen for the tests. Here, the average size of clusters was measured as 12.5 µm in radius. Other parameters were the same as for the previous study at a CW laser power of 180 mW. The speeds of beads located 75 µm away from the beam centre are plotted as a function of bead size in Fig. 5f. The average bead speed increases with size except for particles with *Φ*1.54 µm, a result which can be explained in terms of the suspension position being higher for the smallest beads as follows. The suspension heights for the five different particle sizes were determined by measuring the vertical displacement between the geometric central points of the particle bodies and the top surface of the substrate by moving the focal plane of the microscope. The vertical positions of the particles were plotted as a function of particle size, as shown in Fig. 5g where the error bars represent the standard deviations of the measurements. The vertical suspension position increases with bead diameter, suggesting the beads are sedimenting to a position close to the substrate, except for the case of the smallest beads whose position is the highest of the first three bead sizes and are therefore experiencing some buoyancy. Figure 5h displays the modelled flow speed at the corresponding suspension positions for two different SWNT cluster sizes. The results are remarkably close to the experimental results of Fig. 5f. Therefore, it can be concluded that the bead speed was determined by its height where it matched the local speed of the laminar fluid flow. In addition, it also can be seen from Fig. 5h that particles tend to move faster with smaller SWNT cluster except for the largest particle size, in which the two data points almost overlap.

According to the results in Fig. 4c, the particle-stopping position increases with particle size. In this case, the particles stop moving when they experience two balanced forces that can also be considered as two velocities with the same magnitude but opposite directions. The convection flow speeds and the temperature gradients can be obtained from the simulation. It is therefore possible to determine the relation between the thermophoretic mobility ($D_{T}$) and particle size. The particle-stopping position in three dimensions can be identified based on Fig. 4c and Fig. 5g. Using the relation between the thermophoretic velocity and $D_{T}$ (Eq. (2)), the thermophoretic mobility as a function of particle size is displayed in Fig. 5i. It is concluded that the thermophoresis is unambiguously thermophobic, with $D_{T}$ increasing with particle size and following a nearly linear trend. This behaviour agrees with some reports in the literature^30,38,45^. Here, the simulations were all performed with an input power of 10 mW.

### Cell sample

The simplicity of implementing this manipulation mechanism suggests it may prove useful for concentrating biological samples, as it requires only the addition of SWNTs to the solutions and standard glassware can be used without modification. To assess the feasibility of concentrating cells, a Jurkat cell line was used in the following experiment.

First it was essential to establish that introducing SWNT clusters into the sample solution has no impact on cell viability. This was done by introducing 0.4% Trypan blue stain and following the standard protocol^57^. For this experiment, the SWNT cluster solution was added to a culture flask containing cells, and the flask was then replaced in the incubator. The total number of cells and dead cells were counted every 30 min in the first 3 h and then every hour for a further 2 h. Almost 97% of cells were alive after 5 h, which indicates good biocompatibility of the SWNT cluster solution. It was concluded cell viability is unlikely to be affected by a microfluidic device containing SWNT clusters.

The microfluidic device was sterilized under UV light exposure for 2 h before loading with a sample solution containing Jurkat cells and SWNT clusters.

Cell manipulation experiments were carried out using the experimental system and conditions as previously. Figure 1c,d show the first and last images of the video taken during the experiment, with cells accumulating around the cluster, demonstrating that cells can be trapped and aggregated using our device in the same way as microbeads in the earlier experiments. The sample solution also contained microbeads for indicator purposes only (see Supplementary Video S3 for the real-time video).

Further investigation was undertaken of the temperatures experienced by cells within the ring region. The temperatures were modelled for the cell positions in Fig. 1d, where the average temperature was found to be 67 °C. Song et al*.*^58^ have reported that, after continuous heating for 5 min, human prostate cancer cells were fated to undergo cell death at 51 °C. Therefore, the conditions in our experiment will not be favourable for cell viability. In order to maintain a healthy and biocompatible environment, our system needs to be operated at a lower optical power to keep the cell temperature below 51 °C. We therefore modelled the ambient temperature around cells for different absorbed optical powers. The results are summarized in Table 2. At 6 mW, the average ambient temperature is around 49 °C, which is 2 °C below the temperature threshold of 51 °C and therefore sufficiently low to ensure cell survival. Compared to an absorbed optical power of 10 mW, the flow speeds decrease, so a longer time would be required to form the ring region, and the particle-stopping positions may be affected as well.

**Table 2:** Modelled averaged ambient temperature around cells, and flow speed at 75 µm from the beam centre for different absorbed optical powers.

Optical power absorbed by SWNT cluster (mW)	Averaged ambient temperature around cells (°C)	Flow speed at 75 µm from beam centre (µm s^−1^)
10	67	1.2
6	49	0.7
5	45	0.6

## Conclusion

In summary, we have developed an alternative way to move microparticles with optical-thermophoretic tweezers using a combination of thermal convention and thermophobic thermophoresis. We show both micron-sized polymer beads and cells can be manipulated and form a ring region around the laser heating region due to two competing forces: the Stokes drag force and the thermophoretic force. Experimental results and numerical modelling show that the Stokes drag force increases linearly with light absorption. In a microfluidic chamber both microparticles and cells settle close to the substrate, so the Stokes drag force increases with particle size reflecting the laminar flow distribution of the convection current. The thermophoretic force also increases with particle size, suggesting the technique could be used in size sorting applications. Compared to other tweezing techniques (such as optical tweezers^11,12^, acoustic tweezers^22–24^, etc.), this system uses optical components with low numerical aperture (NA), which do not require a complex optical system aligned with high accuracy. Furthermore, the optical intensities are relatively low (< 2 × 10^5^ Wcm^‒2^) compared to other optical techniques. Although the temperatures produced in some of the experiments would impact cell viability, these conditions could be used for applications where cell viability is not important such as the concentration of sparse cells for identification. Alternatively, the power (and hence temperature rise) can be reduced to ensure the technique is not harmful to biological samples. The device is cheap, disposable, easy to fabricate and use, and does not require any patterning on the substrate surface. Furthermore, it is straightforward to prepare the sample solution.

While this technique is still in its early stages, we believe that by integrating established techniques into the system it will become a useful and versatile tool for researchers in bio-chemical, biological and biomedical fields. For instance, by using spatial light modulator (SLM) and a normal microscope slide with sufficient CNTs added to the solution, different light patterns (such as rings, parallel lines, etc.) could be constructed, making it possible to trap and move single or multiple particles or biological molecules freely. In addition, the optical powers could be reduced by increasing the areal density of the SWNTs and reducing the gaps between them. Given that only 10 mW from an incident optical power of 180 mW is absorbed in the present system, a highly dense CNT sheet could reduce the required optical power by a factor of up to 18.

## Supplementary information

Supplementary Information.

Supplementary Video S1.

Supplementary Video S2.

Supplementary Video S3.
