# Supplementary material for: Microparticle manipulation using laser-induced thermophoresis and thermal convection flow
Source: Sci Rep. 2020 Nov 5;10:19169. doi: 10.1038/s41598-020-76209-9 (PMC7644619; doi:10.1038/s41598-020-76209-9)
Supplement: Supplementary file 1 — Supplementary Information. [file 41598_2020_76209_MOESM1_ESM.docx]

**Supplementary Information**

**Microparticle manipulation using laser-induced thermophoresis and thermal convection flow**

Yang Qian^1^, Steven L. Neale^1^ and John H. Marsh ^1,*^

^1^ School of Engineering, University of Glasgow, G12 8QQ, UK.

^*^ E-mail: [John.Marsh@glasgow.ac.uk](mailto:John.Marsh@glasgow.ac.uk)

**Supplementary Videos**

**Video S1:** Manipulation process of 1.54 μm PS beads with a SWNT cluster using laser-induced thermal forces.

**Video S2:** Demonstration of laser-induced thermal convection flow with a SWNT cluster (sample: 1.54 µm PS beads).

**Video S3:** Manipulation process of Jurkat cells and 1.54 μm PS beads with a SWNT cluster using laser-induced thermal forces.

**Supplementary figure**

Simulation

3D modeling under steady-state conditions based on the experiments was performed using COMSOL Multiphysics modeling software. The 3D geometric structure used to represent the sample device is shown in Fig. S1. The structure was constructed from four parts: the cover slide domain (representing the glass cover slide), the water domain (representing the closed fluid chamber), the heat source domain (representing the central area of laser heating within the SWNT cluster) and the substrate domain (representing the glass substrate). Regarding the real thicknesses of the device, the total size of the structure (*L* × *W* × *H*) was 1 mm × 1 mm × 1.6 mm. The heat source domain was built as a cylinder within the water domain with its bottom resting on the top surface of the substrate domain. Its dimensions (*R* × *H*) were set based on the average measurements of the SWNT clusters, which were 12.5 µm and 31 µm.

The laser-induced thermal convection flow was modeled by coupling the heat transfer model and the incompressible laminar flow model. In the heat transfer model, the type for the water domain was set as ‘fluid’ and the remainder of the domains were set as ‘solid’. Heat transfer was calculated according to the heat equation

$$\rho C_{p}\frac{\partial T}{\partial t}+\rho C_{p}u\cdot\nabla T+\nabla\cdot q=Q$$

(1)

where *q* = *-k*∇*T*, *ρ* is the density, *C_p_* is the specific heat capacity at constant pressure, *T* is the absolute temperature, *u* is the velocity field, *k* is the thermal conductivity and *Q* is the heat source. The heat source domain was set to be a body heat source (*Q*_0_) which can be expressed as

$$Q=Q_{0}=\frac{P_{0}}{V}$$

(2)

where *P*_0_ is the input power in Watts and *V* is the total volume of the selected domain. In our case, the input power should be the absorbed optical power, which was measured as 10 mW. For the boundary conditions, a convective heat flux was present at the exterior surfaces of the cover slide and the substrate domains to simulate natural convection cooling. The ‘diffuse surface’ boundary condition was applied to the water-solid and air-solid interfaces, along with the corresponding surface emissivity, which accounted for heat transfer by radiation between surfaces. In addition, the initial temperatures of all domains were set as 20 °C.

In terms of the laminar flow model, an incompressible flow approximation was used which is valid for small changes in temperature and allows density changes to be accounted for by a volume force. By activating the gravity feature, the fluid density-dependent volume force of buoyancy was automatically added in the momentum equation in order to simulate the buoyancy-driven convection flow. The initial flow velocity was set to 0 to simulate the initial condition of the experiment. Therefore, based on above conditions, the fluid flow was analyzed according to the Navier-Stokes equation

$$\rho_{ref}\left(u\cdot\nabla\right)u=\nabla\cdot\left[-pI+\mu_{ref}\left(\nabla u+\left(\nabla u\right)^{T}\right)\right]+F$$

(3)

$\rho_{ref}\nabla\cdot\left(u\right)=0$, $\mu_{ref}=\mu\left(T_{ref}\right)$, $F=-\rho_{ref}\alpha_{p}\left(T_{ref}\right)\left(T-T_{ref}\right)g$, $\alpha_{p}\left(T_{ref}\right)=-\frac{1}{\rho}\left.\frac{\partial\rho}{\partial T}\right|_{T_{ref}}$

(4)

where *ρ_ref_* is the reference density, *p* is the pressure, *I* is the identity matrix, *µ* is the dynamic viscosity, *T_ref_* is the reference temperature and *F* is the volumatic force. For the boundary conditions, the surfaces in contact with the water domain and the sidewalls of the domain were set as ‘no slip’, which means the flow velocity immediately adjacent to a wall is 0.


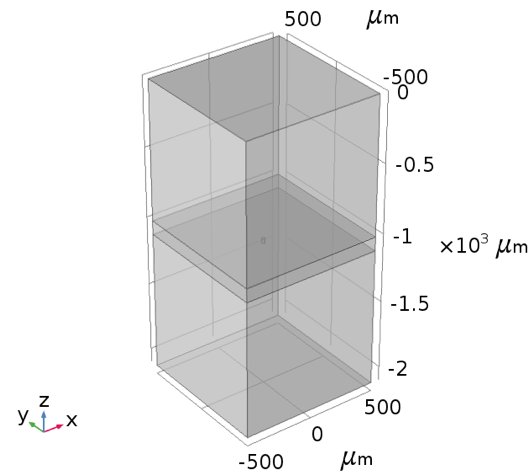


Cover slide domain

Water domain

Substrate domain

Heat source (laser heating) domain

**b**

**a**

Convection flow

Glass

Glass

Laser beam

Air layer

Heat sink

SWNT cluster

**Figure S1.** Schematics of (a) the sample device configuration (cross section view) and (b) the 3D structure built in the simulation based on the actual thickness of the sample device. Here, figure (a) was created by Microsoft Word v15.29, URL: https://www.microsoft.com/en-gb/microsoft-365/word; figure (b) was created by COMSOL v5.3, URL: https://uk.comsol.com/.

Modelled results of control experiment

**a**


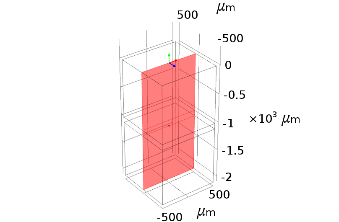

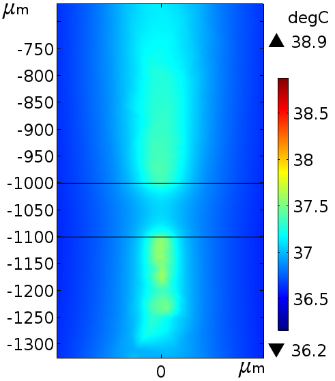

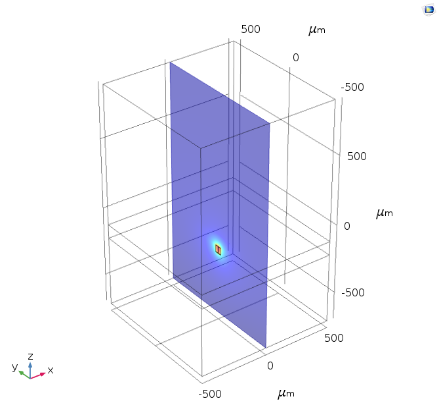


*x-z* cross-section

Cover slide domain

Water domain

Substrate domain


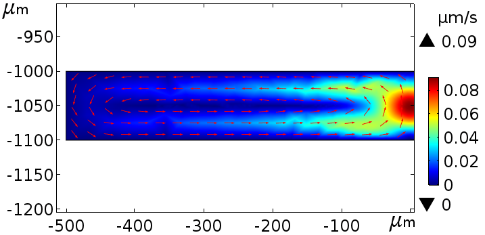


*x-z* cross-section

**b**

**Figure S2**. Numerical modeling of control experiment based on glass substrate system without SWNT clusters. (a) Temperature distributions within the 3D structure in an *x-z* cross-section. (b) Flow velocity magnitude distribution (shown as color background) and direction (red arrows) within the water domain of the structure in an *x-z* cross-section.
